# Substitution of ethambutol with linezolid during the intensive phase of treatment of pulmonary tuberculosis: study protocol for a prospective, multicenter, randomized, open-label, phase II trial

**DOI:** 10.1186/s13063-017-1811-0

**Published:** 2017-02-13

**Authors:** Ji Yeon Lee, Deog Kyeom Kim, Jung-Kyu Lee, Ho Il Yoon, Ina Jeong, Eunyoung Heo, Young Sik Park, Jae Ho Lee, Sung Soo Park, Sang-Min Lee, Chang-Hoon Lee, Jinwoo Lee, Sun Mi Choi, Jong Sun Park, Joon-Sung Joh, Young-Jae Cho, Yeon Joo Lee, Se Joong Kim, Young Ran Hwang, Hyeonjeong Kim, Jongeun Ki, Hyungsook Choi, Jiyeon Han, Heejung Ahn, Seokyung Hahn, Jae-Joon Yim

**Affiliations:** 1 Division of Pulmonary and Critical Care Medicine, Department of Internal Medicine, National Medical Center, Seoul, Republic of Korea; 2 Division of Pulmonary and Critical Care Medicine, Department of Internal Medicine, Seoul National University Boramae Medical Center, Seoul, Republic of Korea; 3 Division of Pulmonary and Critical Care Medicine, Department of Internal Medicine, Seoul National University Bundang Hospital, Seongnam, Republic of Korea; 4 Division of Pulmonary and Critical Care Medicine, Department of Internal Medicine, Seoul National University Hospital, Seoul, Republic of Korea; 5 Medical Research Collaborating Center, Seoul National University Hospital, Seoul, Republic of Korea; 6 Department of Internal Medicine, Seoul National University College of Medicine, Seoul, Republic of Korea

**Keywords:** Tuberculosis, Multicenter, Randomized trial, Ethambutol, Linezolid

## Abstract

**Background:**

Linezolid, an oxazolidinone, substantially improves treatment outcomes of multidrug-resistant tuberculosis and extensively drug-resistant tuberculosis. We started a trial to test whether the use of linezolid instead of ethambutol could increase the rate of sputum culture conversion as of 8 weeks of treatment in patients with drug-susceptible tuberculosis.

**Methods/design:**

This is a phase II, multicenter, randomized study with three arms. We are enrolling patients with pulmonary tuberculosis without rifampicin resistance screened by the Xpert MTB/RIF® assay. The standard treatment arm uses isoniazid (6 months), rifampicin (6 months), pyrazinamide (2 months), and ethambutol (2 months). Experimental arm 1 uses linezolid (600 mg/day) for 4 weeks instead of ethambutol. Experimental arm 2 uses linezolid (600 mg/day) for 2 weeks instead of ethambutol. The primary outcome is the sputum culture conversion rate on liquid media after 2 months of treatment. Secondary outcomes include the sputum culture conversion rate on solid media after 2 months of treatment, time to sputum culture conversion on liquid and solid media, cure rate, and treatment success rate. The frequencies of total adverse events (AEs) and serious AEs will be described and documented. Based on an α = 0.05 level of significance, a power of 85%, a 15% difference in the culture conversion rate after 2 months between the control arm and experimental arm 1 (75% vs. 90%), a 10% default (loss to follow-up) rate, and a 10% culture failure, the required number per arm was calculated to be 143 (429 in total).

**Discussion:**

This trial will reveal the effectiveness and safety of 2 or 4 weeks of use of linezolid instead of ethambutol for patients with drug-susceptible pulmonary tuberculosis. If a new regimen including linezolid shows a higher culture conversion rate by week 8, and is safe, it could be tested as a 4-month antituberculosis treatment regimen in the future.

**Trial registration:**

ClincalTrials.gov, NCT01994460. Registered on 13 November 2013.

**Electronic supplementary material:**

The online version of this article (doi:10.1186/s13063-017-1811-0) contains supplementary material, which is available to authorized users.

## Background

Tuberculosis (TB) is a major challenge for the maintenance of human health. In 2014, there were 9.6 million new cases of TB, including 5.4 million men, 3.2 million women, and 1.0 million children. TB killed 1.5 million people in 2014 [1].

The current standard short-course treatment for pulmonary TB requires 6 months to complete. This long treatment duration increases the likelihood of adverse effects while decreasing patients’ adherence to anti-TB drugs. As many as 16.8% of patients with TB who were treated with the 6-month regimen showed poor compliance [2]. Patients with TB who take their medication irregularly could spread TB to others, and/or their condition could progress to drug-resistant TB. A new, shorter duration anti-TB treatment regimen could decrease the frequency of this poor compliance.

Linezolid, an oxazolidinone, exerts antibacterial activity via inhibiting protein synthesis by binding the 23S ribosomal RNA portion of the bacterial 50S ribosomal subunit [3]. Linezolid has shown modest early bactericidal activity against *Mycobacterium tuberculosis* [4, 5]. However, adding linezolid has resulted in a culture conversion rate of 89% by 6 months in patients with extensively drug-resistant TB (XDR-TB) refractory to previous treatment [6].

Considering its impressive anti-TB effect, linezolid could also have additive activity when administered along with existing anti-TB drugs in patients with drug-susceptible pulmonary TB. We hypothesize that the use of linezolid instead of ethambutol will increase the culture conversion rate by week 8.

## Methods/design

### Setting

**Fig. 1:**
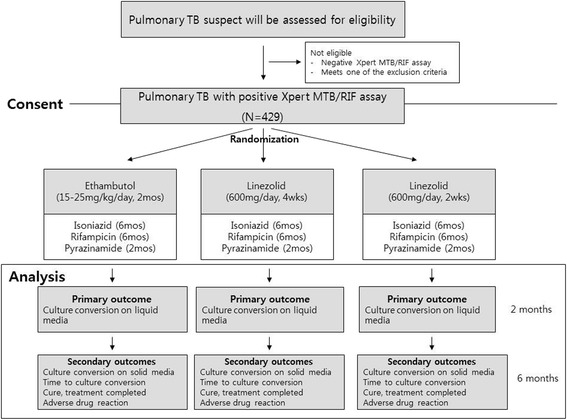
Flow diagram of the trial

This randomized controlled trial is to be conducted at four hospitals in South Korea. The flow diagram of the trial is shown in Fig. 1. Three of the hospitals are affiliated with the Seoul National University College of Medicine (Seoul National University Hospital, Seoul National University Bundang Hospital, and Seoul National University Boramae Medical Center), and the other is the National Medical Center in Seoul. All hospitals are located in an urban area.

### Design

This is a phase II, multicenter, randomized study with three arms. Eligible patients are first screened by safety laboratory testing and using the Xpert MTB/RIF® assay (Cepheid, Sunnyvale, CA, USA) for rifampicin resistance. Patients with TB without resistance to rifampicin are randomized to the following three arms at a 1:1:1 ratio:

1. Arm 1 (control arm): Standard treatment for drug-sensitive pulmonary TB uses isoniazid (6 months), rifampicin (6 months), pyrazinamide (2 months), and ethambutol (2 months) as recommended by the Korean National Guidelines [7] (Table 1).

**Table 1:** Dose and schedule of first-line anti-TB drugs recommended by the Korean National Guidelines [7]

Drug	Dosage			Frequency and timing
	Dose/kg	Recommended dose	Maximal dose	
Isoniazid	5 mg/kg	300 mg/day	300 mg/day	Daily, before meal
Rifampicin	10 mg/kg	450 mg (<50 kg)	600 mg/day	Daily, before meal
		600 mg (≥50 kg)		
Pyrazinamide	20–30 mg/kg	1000 mg (<50 kg)		Daily, before or after meal
		1500 mg (50–70 kg)		
		2000 mg (>70 kg)		
Ethambutol	15–20 mg/kg	800 mg (<60 kg)		Daily, before or after meal
		1200 mg (60–80 kg)		
		1600 mg (>80 kg)
2. Arm 2 (experimental arm 1): Treatment using isoniazid (6 months), rifampicin (6 months), pyrazinamide (2 months), and linezolid is used (600 mg/day, 4 weeks).
3. Arm 3 (experimental arm 2): Treatment using isoniazid (6 months), rifampicin (6 months), pyrazinamide (2 months), and linezolid is used (600 mg/day, 2 weeks).

The study timeline is shown in Table 2. Returned trial product is reconciled at every visit by the research nurse to ensure adherence to the allocated regimen. If a participant misses a scheduled visit, the research nurse makes immediate phone contact. The duty physician makes a decision on discontinuation or modification of the allocated regimen for patients showing clinical deterioration or reporting adverse events associated with the anti-TB treatment. No treatment or intervention is prohibited for the participants, and any ancillary and/or post-trial care is determined by the duty physician.

**Table 2:** Study timeline

Visit	Screening	Baseline visit^a^	Treatment							
Visit number	1	2	3	4	5	6	7	8	9	10
Weeks (w)	−2 w to −1 d	0	1 w	2 w	4 w	8 w	12 w	16 w	20 w	24 w
Visit window	NA	NA	±4 d	±4 d	±1 w	±2 w	±2 w	±2 w	±2 w	±2 w
Consent	X									
Randomization		X								
Medical history	X									
Physical examination	X		X	X	X	X	X	X	X	X
Neurologic examination	X		X	X	X	X	X	X	X	X
Xpert MTB/RIF® assay	X									
Sputum AFB smear	X	X^d^	X	X	X	X	X	X	X	X
TB culture (solid)	X	X^d^	X	X	X	X	X	X	X	X
TB culture (liquid)	X	X^d^	X	X	X	X	X	X	X	X
DST^b^	With first cultured *Mycobacterium tuberculosis*									
CXR	X	X^d^			X	X	X	X	X	X
HIV, HBV	X									
Total bilirubin, AST/ALT, creatinine	X	X^d^	X	X	X	X	X	X	X	X
Complete blood count	X	X^d^	X	X	X	X	X	X	X	X
Optic test		X	X	X	X	X				
Urine HCG^c^	X									
Compliance of drug intake			X	X	X	X	X	X	X	X
Adverse drug reaction			X	X	X	X	X	X	X	X
Other medications^e^		X	X	X	X	X	X	X	X	X

*w* weeks, *d* days, *NA* not applicable, *AFB* acid-fast bacilli, *TB* tuberculosis, *DST* drug susceptibility test, *CXR* simple chest radiography, *HIV* human immunodeficiency virus, *HBV* hepatitis B virus, *AST* aspartate transaminase, *ALT* alanine transaminase, *HCG* human chorionic gonadotropin

^a^Treatment will be initiated at the baseline visit, as TB treatment should be started promptly following diagnosis

^b^Drug susceptibility test for isoniazid, rifampicin, ethambutol, pyrazinamide, streptomycin, kanamycin, amikacin, capreomycin, ofloxacin, levofloxacin, moxifloxacin, prothionamide, cycloserine, and *para*-aminosalicylic acid

^c^Only in females of childbearing potential

^d^Can be omitted if the results of previous tests within the prior 4 weeks are available

^e^In particular, immunosuppressive agents (including corticosteroids)

### Outcomes

The primary outcome is the proportion of participants with sputum culture conversion on liquid media after 2 months of treatment. Secondary outcomes include the proportion of participants with sputum culture conversion on solid media after 2 months of treatment, the interval from enrollment to sputum culture conversion on liquid and solid media, the proportion of cured participants, and the proportion of participants with a treatment success rate.

### Definitions

#### Culture conversion and rate of culture conversion

We define culture conversion as two consecutive negative sputum cultures on liquid or on solid media. The date of culture conversion is defined as the date of the initial negative culture. Negative sputum cultures followed by contaminated cultures are also regarded as culture conversion. Culture conversion is also defined as a case where the participant cannot expectorate sputum after one negative sputum culture.

#### Treatment outcomes

We define treatment outcomes according to the Korean TB Guidelines as follows [7]:

- Cure: We define cure as a negative sputum culture after (or during the last months of) treatment combined with one or more prior negative sputum cultures.
- Treatment completed: We define treatment completed as (1) a negative sputum culture after or at the end of treatment, but without a prior negative sputum culture or (2) a negative sputum culture during treatment, but the lack of a negative sputum culture after or during the last month of treatment.
- Treatment success: We define treatment success as achievement of a cure or treatment completion.

### Eligibility criteria for participants

Participants with pulmonary TB satisfying the inclusion criteria are enrolled competitively by investigators in the outpatient clinic as well as the inpatient setting in the four participating hospitals. The inclusion criteria are as follows: men and women aged from 20 to 80 years, with documented sputum Xpert MTB/RIF® assay-positive pulmonary TB at screening, and administration of current TB therapy (if any) for ≤7 days at the time of enrollment.

Patients will be excluded if they have HIV/AIDS. Female patients of childbearing potential who are pregnant, breastfeeding, or unwilling to avoid pregnancy will also be excluded. Additionally, any of the following factors will lead to exclusion: (1) absolute neutrophil count of <2000 cells/mL, (2) white blood cell count of <3.0 × 10^3^/μL, (3) hemoglobin levels of <7.0 g/dL, (4) serum creatinine levels of >2.0 mg/dL, (5) aspartate aminotransferase levels of >100 IU/L, (6) alanine aminotransferase levels of >100 IU/L, (7) total bilirubin levels of >2.0 mg/dL, (8) history of optic neuritis or peripheral neuropathy, and (9) other significant laboratory abnormalities (i.e., absolute neutrophil count, creatinine levels). Finally, we also exclude patients with the need for ongoing therapy with selective serotonin reuptake inhibitors, tricyclic antidepressants, serotonin 5-hydroxytrptamine 1 receptor agonists (triptans), meperidine, buspirone, monoamine oxidase inhibitors, sympathomimetic agents (e.g., pseudoephedrine), vasopressive agents (e.g., epinephrine, norepinephrine), or dopaminergic agents (e.g., dopamine, dobutamine).

### Randomization

We assign participants to study arms using an adaptive stratified sampling method to minimize the imbalance between the numbers of participants in each treatment group over a number of stratification factors: the institution, the presence/absence of cavitation on baseline chest radiographs, and the presence/absence of baseline diabetes mellitus. A centralized web-based system of the Medical Research Collaborating Center of Seoul National University Hospital is used for allocation.

Diabetes mellitus is defined by any one of the following: (1) fasting plasma glucose levels ≥126 mg/dL, (2) random plasma glucose levels ≥200 mg/dL, or (3) the presence of any antidiabetic agent as a concomitant medicine.

### Justification of sample size

The hypothesis of this study is that the use of linezolid instead of ethambutol will increase the sputum culture conversion rate on liquid media after 2 months of anti-TB treatment. A primary comparison will be performed between the control arm and experimental arm 1 (linezolid, 600 mg/day for 4 weeks).

#### Hypothesis for sample size calculation

Ho (null hypothesis): *p*_t_ = *p*_c_ (culture conversion rate after 2 months of treatment is not different between the control arm and experimental arm 1 [linezolid for 4 weeks]).

H_1_ (alternative hypothesis): *p*_t_ ≠ *p*_c_ (culture conversion rate after 2 months of treatment is different between the control arm and experimental arm 1 [linezolid for 4 weeks]).

#### Assumptions

The sputum culture conversion rate on liquid media after 2 months in patients who are treated at Seoul National University Hospital is approximately 75% [8]. We assume that there will be a 90% culture conversion rate on liquid media after 2 months in experimental arm 1 (primary hypothesis) and a 10% default (loss to follow-up) rate, and that 10% of participants will have a positive Xpert MTB/RIF® assay result but a negative result for *M. tuberculosis* culture.

Based on an α = 0.05 level of significance, a power of 85%, and a 15% difference in the culture conversion rate after 2 months between the control arm and experimental arm 1 (75% vs. 90%), the sample size per arm was calculated to be 114 (342 in total). After consideration of a 10% default rate and 10% culture failure in participants with a positive Xpert MTB/RIF® assay result, the final number per arm was calculated to be 143 (429 in total).

### Statistical analysis

The results of this trial will be analyzed based primarily on the intention-to-treat (ITT) approach. In addition, a per protocol analysis will be performed secondarily. A safety analysis will be performed based on the safety group. The ITT groups will comprise participants who satisfy the inclusion and exclusion criteria, those who are randomized, and those who take the trial drug at least once. The per protocol groups will comprise participants from ITT satisfying the following conditions: (1) participants took the trial drug (ethambutol or linezolid) at greater than 80% of the planned dose (ethambutol, ≥45 days for control arm; linezolid, ≥23 days for experimental arm 1; linezolid, ≥12 days for experimental arm 2), and (2) participants completed the clinical trial according to the protocol. The safety group includes participants who took the trial drug at least once. No interim analysis of the data is planned.

### Efficacy outcomes

Comparisons will be performed using two-tailed tests with a statistical significance of 5%. A sensitivity analysis will be carried out based on participants with no resistance to rifampicin, isoniazid, ethambutol, or pyrazinamide. The primary comparison will be performed between the control arm (arm 1) and experimental arm 1 (arm 2). The comparison between the control arm and experimental arm 2 (arm 3) is solely investigational.

#### Analysis of primary outcome

For the primary outcome of this trial, a negative culture conversion rate on liquid media after 2 months of treatment, the culture conversion rate will be estimated as the proportion with a 95% confidence interval for each treatment group. The difference between the control arm and experimental arm 1 (linezolid for 4 weeks) will subsequently be determined using the chi-square test or Fisher’s exact test.

#### Analysis of secondary outcomes

The analysis of secondary outcomes will be described as explorative outcomes. The sputum culture conversion rate after 2 months of treatment (solid media) among the three groups will be compared using the chi-square test or Fisher’s exact test. The median time to culture conversion will be calculated in each group and compared using a log-rank test. Cure rate, treatment completion rate, and treatment success rate will be compared among the three groups using the chi-square test or Fisher’s exact test.

### Safety assessment

Grades 1 and 2 peripheral or optic neuropathies and any grade 3 adverse events (AEs), according to the Common Terminology Criteria for Adverse Events, that are considered possibly, probably, or definitely related to the research, and all serious AEs, will be fully and completely documented.

A safety analysis will be performed based on AEs that are identified during the clinical trial. Frequencies and fractions of total AEs, serious AEs, and AEs will be described, together with 95% confidence intervals. In addition, the relatedness of AEs to the trial drug and their seriousness will be summarized. Frequencies of grade 3 or 4 AEs and those of each AE will be compared using the chi-square test or Fisher’s exact test.

### Stratified analysis

Primary and secondary outcomes will be analyzed separately in participants with sputum smear-positive and smear-negative pulmonary TB.

#### Data collection and management

This study will use a web-based electronic case report form (eCRF) with Pharmaco-epidemiology and Clinical Trial Application X (PhactaX). PhactaX has been developed by the Medical Research Collaborating Center of Seoul National University Hospital in collaboration with an outsourced contractor. PhactaX is based on Java and Oracle databases, in compliance with international standards and regulations. The eCFR designed for this study used dummy variables for user acceptance testing to confirm its validity.

During the study, the field monitor will visit the sites every 3 months to check the completeness of patient records, accuracy of eCRF entries, adherence to the protocol and to Good Clinical Practice, and enrollment progress, and to ensure the correct storage, dispensing, and accountability of the trial products.

#### Supervision of the trial

A data and safety monitoring board will consist of two unblinded independent pulmonologists and one unblinded biostatistician. Based on data review during the trial conduct, the board may provide recommendations such as protocol amendment, continuation, or stopping of the trial.

#### Confidentiality

We will collect the protected health information of study participants only when necessary to evaluate the efficacy, safety, and tolerability of the study medication used in this trial. Under privacy rules and relevant data security rules and safety guidelines, the investigators will carefully collect and use the protected health information of study participants. Paper files containing participants’ data (including personally identifiable information and copies of the signed consent forms) will be securely stored in a locked office on campus in locked filing cabinets. Digital files containing participants’ data will be stored in password-protected files on university-maintained servers. Access to study files will be restricted to authorized personnel only.

**Fig. 2:**
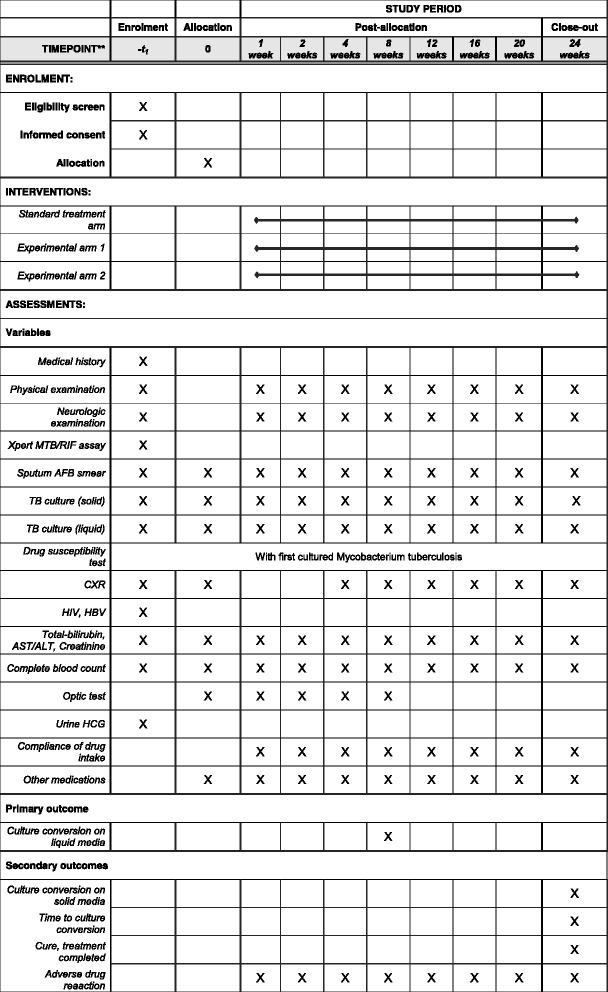
Completed SPIRIT figure

The items in the present study protocol comply with the Standard Protocol Items: Recommendations for Interventional Trials (SPIRIT) checklist (see the SPIRIT Checklist and figure in Additional file 1 and Fig. 2).

## Discussion

Development of a shorter duration anti-TB treatment regimen could improve not only treatment outcomes of individual patients but also TB control throughout the world. Based on higher culture conversion rates after 2 months of treatment in drug-susceptible TB patients treated with regimens, including moxifloxacin [9, 10], 4-month regimens, including moxifloxacin or gatifloxacin, have been attempted recently. The REMoxTB trial replaced isoniazid or ethambutol with moxifloxacin [11]. The OFLUOTUB trial used gatifloxacin instead of ethambutol [12]. The RIFAQUIN trial included moxifloxacin instead of isoniazid in the intensive phase and used twice-weekly application of moxifloxacin and rifapentine in the continuation phase [13]. Another trial tested three-times-weekly administration of isoniazid, rifampicin, pyrazinamide, and moxifloxacin or gatifloxacin instead of ethambutol [14]. Unfortunately, none of these 4-month treatment regimens showed non-inferiority to a 6-month regimen.

Based on the impressive effectiveness of linezolid in patients with XDR-TB refractory to previous treatment [6], we are performing a trial using linezolid instead of ethambutol for drug-susceptible pulmonary TB. If a new regimen including linezolid shows an excellent culture conversion rate by week 8, which could be a surrogate for relapse [15, 16], it could be tested as a 4-month anti-TB treatment regimen through another clinical trial.

Safety issues of using linezolid for TB treatment will also be addressed through the current trial. Although linezolid is highly effective for the treatment of multidrug-resistant TB, it is frequently accompanied by serious AEs, including peripheral neuropathy and bone marrow suppression. In a recent clinical trial of linezolid in patients with XDR-TB, as many as 87% of patients reported clinically significant AEs [6]. Similarly, a meta-analysis reported AEs in 58.9% of multidrug-resistant or XDR-TB patients who used linezolid [17]. These reports of frequent AEs underscore the limitation of linezolid as a standard drug for treatment of multidrug-resistant TB, which mandates the long-term use of anti-TB drugs. Our trial using linezolid for 2 or 4 weeks, times which are within the permitted duration of use, will reveal the safety of the relatively short-term use of linezolid for patients with TB.

Finally, several issues should be considered once the results of this trial become available. First, this trial is being performed only in referral hospitals. The characteristics of patients with TB diagnosed and treated in referral hospitals could differ from those of patients treated in community hospitals. Second, we exclude HIV/AIDS patients from this trial. The application of trial results in areas with a high burden of HIV/AIDS should, therefore, be approached with caution. Third, this is an open-label trial, so the possibility of bias should be taken into account when interpreting results. Finally, if the usefulness of linezolid for drug-susceptible TB is confirmed through this study, strategies to address issues of cost and availability should be examined.

### Trial status

Recruitment began at the first site in February 2014 and is expected to be completed by July 2017.

## Additional file

Additional file 1: SPIRIT checklist. (DOC 126 kb)

## Abbreviations

AE: Adverse event
eCRF: Electronic case report form
ITT: Intention to treat
PhactaX: Pharmaco-epidemiology and Clinical Trial Application X
TB: Tuberculosis
XDR-TB: Extensively drug-resistant TB
